# Taxonomy and Phylogeny of Eight New *Acrophialophora* Species (Sordariales, Chaetomiaceae) from China

**DOI:** 10.3390/jof9060645

**Published:** 2023-06-05

**Authors:** Lan Peng, Yan-Wei Zhang, Hai-Yan Wang, Chun-Bo Dong, Wan-Hao Chen, Jian-Dong Liang, Yan-Feng Han

**Affiliations:** 1 Institute of Fungus Resources, Department of Ecology/Key Laboratory of Plant Resource Conservation and Germplasm Innovation in Mountainous Region (Ministry of Education), College of Life Sciences, Guizhou University, Guiyang 550025, China; 2 School of Biological Sciences, Guizhou Education University, Guiyang 550018, China; 3 Center for Mycomedicine Research, Basic Medical School, Guizhou University of Traditional Chinese Medicine, Guiyang 550025, China; cwhisaria@163.com (W.-H.C.); cordyceps@yeah.net (J.-D.L.)

**Keywords:** Ascomycota, morphology, multi-gene phylogeny, new species, taxonomy

## Abstract

The genus *Acrophialophora* belongs to the family *Chaetomiaceae*. With the addition of new species and transferred species from other genera, the genus *Acrophialophora* has expanded. In this study, eight new species related to *Acrophialophora* were isolated from soil samples in China. Using muti-locus phylogenetic (ITS, LSU, *tub2* and *RPB2*) analysis combined with morphological characteristics, eight new species (*Acrophialophora curvata*, *A. fujianensis*, *A. guangdongensis*, *A. longicatenata*, *A. minuta*, *A. multiforma*, *A. rhombica*, and *A. yunnanensis*) are described. Descriptions, illustrations, and notes of the new species are also provided.

## 1. Introduction

The genus *Acrophialophora* was established by Edward, with *A. nainiana* as the type species, in 1959 [1]. However, the genus was similar to *Paecilomyces* in morphology [2]. In 1968, Dal and Peyronel considered that *A. nainiana* may be a synonym for *Paecilomyces fusisporus*, as they have similar characteristics of the chain conidia and their development from phialides [3]. Despite these similarities, there are important differences that make this genus distinct from *Paecilomyces*. In the subsequent study of Samson and Mahmood in 1970, *P. fusisporus* was transferred to *Acrophialophora*. Then, based on the three representative species (*A. fusispora*, *A. levis*, and *A. nainiana*), *Acrophialophora* was reintroduced as a thermotolerant genus, and the differences from the *Paecilomyces* species were emphasized [4]. Then, through a phylogenetic analysis of small subunit nuclear rRNA gene (SSU) sequences, Luangsa-ard et al. demonstrated that the morphological concept of *Paecilomyces* was polyphyletic [5].

Brown and Smith, in their study on the genus *Paecilomyces*, first introduced one monophialidic species, *P. inflatus*, in 1957 [6]. Then, Onions and Barron transferred 10 monophialidic species, including *P. inflatus*, to the genus *Paecilomyces* in 1967 [7]. Gams transferred other awl-shaped monophialidic species to the genus *Acremonium* and left only *P. inflatus* in the genus *Paecilomyces* in 1971 [8]. Later, many more monophialidic species were described in *Paecilomyces*, such as *P.biformis*, *P. curticatenatus*, and *P. major* [9,10,11,12].

Through a phylogenetic analysis combining the nuclear ribosomal internal transcribed spacer (ITS) and SSU sequences, Liang et al. established the thermotolerant genus *Taifanglania* with *T. hechuanensis* as the type species and transferred eight monophialidic *Paecilomyces* species: *P. ampullaris*; *P. ampulliphorus*; *P. biformis*; *P. cinereus*; *P. curticatenatus*; *P. furcatus*; *P. inflatus*; and *P. major* [13]. Subsequently, three new species, *T. berberidis*, *T. jiangsuensis*, and *T. parvispora*, were added to *Taifanglania* [14,15]. In 2013, *T. inflata* was transferred to *Phialemonium* by Perdomo et al. [16]. Until that time, there were 12 species in the genus *Taifanglania*.

In 2015, Zhang et al. assessed the relationship between *Acrophialophora* and *Taifanglania* through phylogenetic analyses of ITS sequences and combined β-tubulin (*tub2*) and SSU, and they considered *Taifanglania* to be synonymous with *Acrophialophora* and accordingly transferred all *Taifanglania* species to *Acrophialophora* [17]. In addition, three new species were proposed: *A. acuticonidiata*, *A. angustiphialis*, and *A. ellipsoidea* [17]. Subsequently, *Acrophialophora* accommodated more species. Sandoval et al. transferred *Ampullifera seudatica* to *Acrophialophora* and classified *Acrophialophora* in *Chaetomiaceae* [18]. Zhang et al. added the new species *Acrophialophora liboensis* in 2017 [19]. Wang et al. added the new species *Acrophialophora teleoafricana*, and they transferred *Chaetomium jodhpurense* to *Acrophialophora* as *A. jodhpurense* in 2019. Moreover, *A. teleoafricana* and *A. jodhpurense* are the only two species in the genus for which a sexual state is known [20]. Based on the results of a multigene phylogenetic analysis and molecular dating analyses, combined with the morphological and temperature-growth characteristics, 50 genera and 275 species are accepted in *Chaetomiaceae* [21]. To date, the genus *Acrophialophora* includes a total of 20 species. These research results indicate that the genus *Acrophialophora* and its species need a complete and updated taxonomic classification system.

Currently, the development and applications of *Acrophialophora* spp. Have attracted wide attention. *Acrophialophora* is a thermotolerant soil fungus that is widely distributed in temperate and tropical regions [18]. Some species of fungi in nature can withstand high temperatures (above 40 °C). Thermotolerant fungi are those that can grow at a minimum temperature of below 20 °C and a maximum temperature of around 50 °C [22]. Given its capacity to produce large quantities of cellulases and xylanases, *Acrophialophora* is also commonly isolated as a decomposer of compost and other self-heating substrates [23]. In addition, some species of *Acrophialophora* have been found to be human pathogens. For example, *A. fusispora* is currently recognized as an emerging human opportunistic pathogen that is responsible for cases of keratitis, pulmonary colonization, and infection, as well as devastating cerebral infections that require intensive antifungal therapy [18]. To sum up, the application prospects of *Acrophialophora* in the agriculture, environmental protection, and biotechnology industries, as well as in human health and public safety, need to be further explored.

In a survey of the fungal diversity in soil samples from China, 24 new isolates related to *Acrophialophora* were obtained. Based on phylogenetic and morphological studies, the 24 new isolates represent eight new species. Here, we provide eight new species with descriptions, illustrations, and a phylogenetic tree to show the position of the new species.

## 2. Materials and Methods

### 2.1. Sampling, Fungal Isolation, and Morphology

Soil samples were collected in August 2013 from Guangdong, Yunnan, Fujian, and Hainan provinces, southern China, by Y.F. Han. Fungi of the soil samples were isolated according to the method described by Wang et al. [15]. The purified isolates were transferred to potato dextrose agar (PDA) and oatmeal agar (OA) at 35 °C for 7 days in the dark to observe the macroscopic and morphological development of the colonies [17]. The characterization and measurement of the fungal microscopic characteristics were performed in 25% lactic acid. Images were obtained using an optical microscope (BX53, Olympus, Tokyo, Japan) with differential interference contrast (DIC). Colonies on PDA were incubated after 7 days at 35 °C, and the cultures were placed in 50 °C to produce the dried holotypes. The dried holotypes were conserved in the Mycological Herbarium of the Institute of Microbiology, Chinese Academy of Sciences, Beijing, China (HMAS). Ex-type strains were conserved at the China General Microbiological Culture Collection Center (CGMCC) and the Institute of Fungus Resources, Guizhou University (GZUIFR), and other living strains were conserved at the Institute of Fungus Resources, Guizhou University (GZUIFR, GZAC). The morphological descriptions and names of the new taxa were introduced and deposited in MycoBank (https://www.mycobank.org/, asscessed on 3 December 2022).

### 2.2. DNA Extraction, PCR Amplifications, and Sequencing

The genomic DNA was extracted using the BioTeke Fungus Genomic DNA Extraction Kit (DP2032, BioTeke, Beijing, China), following the manufacturer’s instructions. The ITS region, *tub2*, the D1/D2 domains of the 28S nrDNA (LSU), and partial RNA polymerase II second largest subunit (*RPB2*) genes were amplified by PCR, as described by White et al., Glass and Donaldson, Vilgalys and Sun, and Liu et al., respectively [24,25,26,27]. The purified PCR products were obtained and sequencing was performed by a commercial sequencing service provider (Quintarabio, Wuhan, China). In this study, the new sequences mentioned were uploaded to GenBank (Table 1).

### 2.3. Phylogenetic Analyses

The new sequences were uploaded to BLAST and searched in the GenBank database to determine their most probable related taxa. *Acrophialophora* species and outgroup *Chrysocorona lucknowensis* (CBS 727.71) sequence data (ITS, LSU, *tub2* and *RPB2*) were employed to identify the new species. These sequence data were obtained from GenBank (Table 1). The gene regions were concatenated and aligned for phylogenetic analysis. Lasergene software (version 6.0, DNASTAR) was applied for the assembling and editing of the DNA sequences in this study. The consensus sequences from different primers were trimmed using MEGA7. The maximum likelihood (ML) and Bayesian inference (BI) approaches were carried out for the phylogenetic analyses using PhyloSuite v. 1.16 [28]. The maximum likelihood (ML), the best-fit model of substitution for each locus, was estimated using IQ-TREE’s ModelFinder function based on the corrected Akaike information criterion (AICc) [29,30]. The best-fit evolutionary models of ML and BI analyses of each locus are listed in Table 2. The resulting phylogenetic trees were visualized in FigTree v. 1.1.2 and subsequently edited in Adobe Illustrator 2020.

## 3. Results

### 3.1. Phylogeny Analysis

**Figure 1:**
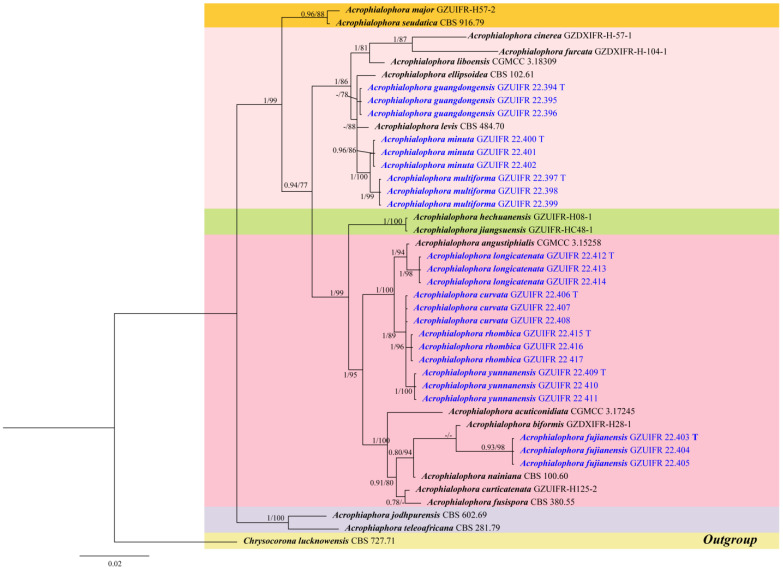
Phylogenetic tree of the genus *Acrophialophora* constructed from ITS, *tub2*, LSU, and *RPB2* gene region alignment. Maximum likelihood bootstrap values ≥ 75% and posterior probabilities ≥ 0.90 are shown above internal branches. The “-” indicates a lack of statistical support. The eight new species are shown in blue. The scale bar shows the expected number of changes per site. Type strains are marked with “T” after the culture number.

Based on a BLAST search (https://blast.ncbi.nlm.nih.gov/Blast.cgi, accessed on 30 July 2022) using the ITS sequences, our isolates were identified as belonging to the genus *Acrophialophora*. To determine the phylogenetic positions of these strains, we performed a multilocus (ITS+*tub2*+LSU+*RPB2*) phylogenetic analysis. The dataset was composed of the ITS (1–530 bp), *tub2* (531–905 bp), LSU (906–1454), and *RPB2* (906–1614 bp) genes, comprising a total of 2163 characters (including gaps). The results showed that the new species belong to the genus *Acrophialophora* (Figure 1). The topologies of the two trees for ML and BI analyses were nearly identical, so we chose BI trees, incorporating ML trees to show the phylogeny. In the phylogenetic tree, eight new species form a well-supported clade separated from other species in *Acrophialophora*, *A. fujiangensis* sp. nov. (0.93 PP/98% BS); *A. curvata* sp. nov. (1 PP/89% BS); *A. yunnanensis* sp. nov. (1 PP/100% BS); *A. longicatenata* sp. nov. (1PP/98% BS); *A. rhombica* sp. nov. (1 PP/96% BS); *A guangdongensis* sp. nov. (- PP/78% BS); *A. multiforma* sp. nov. (1 PP/99% BS); and *A. minuta* sp. nov. (0.96 PP/86% BS).

### 3.2. Taxonomy

The morphological characteristics of eight new species and their illustrations are mentioned below.

**Figure 2:**
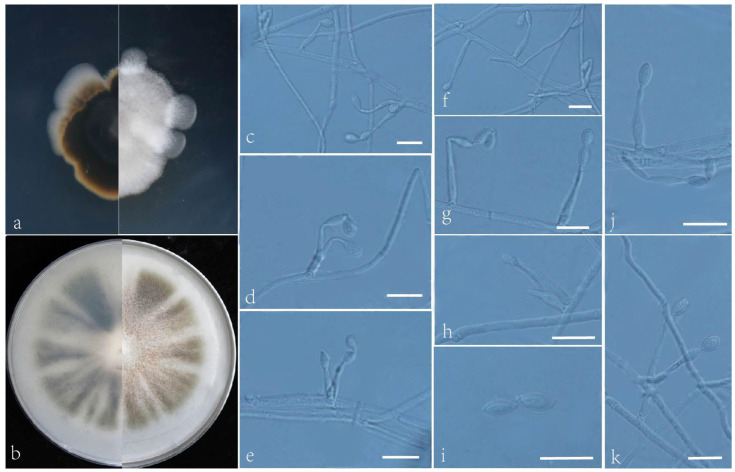
*Acrophialophora curvata* (ex-type CGMCC 3.24167). (**a**,**b**) The reverse and obverse colonies on PDA and OA after 7 d at 35 °C. (**c**–**k**) Hyphae, phialidic conidiogenous cells, and conidia. Scale bars: (**c**–**k**) = 10 μm.

***Acrophialophora curvata*** L. Peng and Y.F. Han sp. nov. Figure 2

MycoBank: 845781

Etymology: Refers to a frequent bend between conidia and phialides.

Holotype: China, Yunnan, Dai Autonomous Prefecture of Xishuangbanna (N 21°40′, E 101°50′), soil, dried holotype HMAS 255324, ex-type culture CGMCC 3.24167 = GZUIFR 22.406. GenBank accession numbers: OP454351 (ITS), OP547306 (*tub2*), OP454363 (LSU), OP802834 (*RPB2*).

Micromorphology: Somatic hyphae hyaline, smooth-walled, septate, and 1–4 μm wide. Conidiophores usually reduced to conidiogenous cells. Conidiogenous cells, phialidic, single, arising laterally from the hyphae, with an obclavate swollen basal portion, tapering into a narrow neck, sometimes proliferating in two furcations, 5–24.5 × 2–2.5 μm. Conidia one-celled, formed in basipetal chains, often composed of one to two conidia, hyaline, smooth, fusiform or ellipsoidal, 4–6.5 × 2–3.5 μm. Sexual morph not observed.

Culture characteristics: Colonies on PDA reaching 34–36 mm diam after 7 d at 35 °C, irregular in the margin, flat, dense, fluffy, gray to white, reverse center to edge, dark, pale brown to gray. Colonies on OA reaching 63–65 mm diam after 7 d at 35 °C, alternating brown and white, bronzing floccose due to aerial mycelium, reverse brown and white.

Geographical distribution: China.

Additional material examined: China, Yunnan, Dai Autonomous Prefecture of Xishuangbanna (N 21°40′, E 101°50′), soil, and our other living strains: GZUIFR 22.407, GZUIFR 22.408. GenBank accession numbers: OP454352 (ITS), OP547307 (*tub2*), OP454364 (LSU), OP802835 (*RPB2*) and OP454353 (ITS), OP547308 (*tub2*), OP454365 (LSU), and OP802836 (*RPB2*).

Notes: Phylogenetically, *Acrophialophora curvata* is closely related to *A. rhombica* (Figure 1); however, the sequence similarity of the *RPB2* between the two species was 98.5%. The features of *A. curvata* are that its phialides frequently proliferate into two, the necks between the phialides and conidia are often curved, and the conidial chain consists of only one or two conidia. These features distinguish it from other species of the genus.

**Figure 3:**
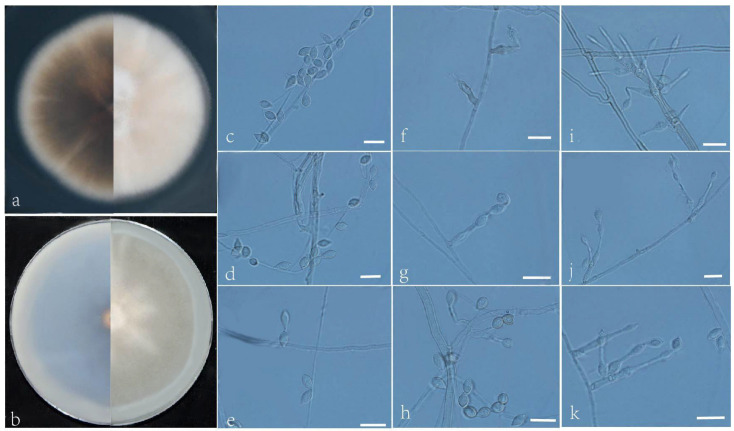
*Acrophialophora fujianensis* (ex-type CGMCC 3.24166). (**a**,**b**) The reverse and obverse colonies on PDA and OA after 7 d at 35 °C. (**c**–**k**) Hyphae, phialidic conidiogenous cells, and conidia. Scale bars: (**c**–**k**) = 10 μm.

***Acrophialophora fujianensis*** L. Peng & Y.F. Han sp. nov. Figure 3

MycoBank: 845782

Etymology: Refers to Fujian Province, where the isolate was collected.

Holotype: China, Fujian, Fuzhou, Fujian Normal University (N 26°03′, E 119°21′), soil, dried holotype HMAS 352279, ex-type culture CGMCC 3.24166 = GZUIFR 22.403. GenBank accession numbers: OP454345 (ITS), OP536984 (*tub2*), OP454372 (LSU), and OP820579 (*RPB2*).

Micromorphology: Somatic hyphae hyaline, smooth-walled, septate, 1–4.5 μm wide. Conidiophores usually reduced to conidiogenous cells. Conidiogenous cells born apically in whorls or in verticils on conidiophores or arising laterally from the hyphae, phialides single, smooth, hyaline, cylindrical or slightly clavate, swollen near the base, tapering to a narrow neck, often proliferating, 8–25 × 2–2.5 μm. Conidia one-celled, formed in basipetal chains, often composed of one to three conidia, hyaline, smooth, fusiform, ellipsoidal or ovoid, 4–9 × 2.5–4.5 μm. Sexual morph not observed.

Culture characteristics: Colonies on PDA reaching 60–65 mm diam after 7 d at 35 °C, mostly regular in the margin, flat, dense, fluffy, beige to white, reverse pale olive, edge white. Colonies on OA reaching 78–80 mm diam after 7 d at 35 °C, flat, fluffy, gray, reverse dark. Sexual morph not observed.

Geographical distribution: China.

Additional material examined: China, Fujian, Fuzhou, Fujian Normal University (N 26°03′, E 119°21′), soil, and our other living strains: GZUIFR 22.404, GZUIFR 22.405. GenBank accession numbers: OP454346 (ITS), OP536985 (*tub2*), OP454373 (LSU), OP820580 (*RPB2*) and OP454347 (ITS), OP536986 (*tub2*), OP454374 (LSU), and OP820581 (*RPB2*).

Notes: Phylogenetically, *Acrophialophora fujianensis* sp. nov. is closely related to *A. biformis* (Figure 1); however, the sequence similarity of ITS between the two species was 98.6%. Morphologically, they can be distinguished by their conidial chains: *A. fujianensis* is often composed of one to three conidia, while *A. biformis* often forms long chains.

**Figure 4:**
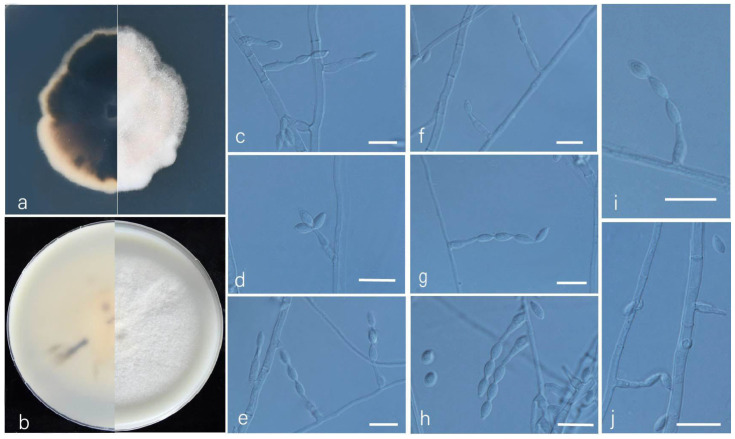
*Acrophialophora guangdongensis* (ex-type CGMCC 3.24163). (**a**,**b**) The reverse and obverse colonies on PDA and OA after 7 d at 35 °C. (**c**–**j**) Hyphae, phialidic conidiogenous cells, and conidia. Scale bars: (**c**–**j**) = 10 μm.

***Acrophialophora guangdongensis*** L. Peng & Y.F. Han sp. nov. Figure 4

MycoBank: 845776

Etymology: Refers to Guangdong Province, where the isolate was collected.

Holotype: China, Guangdong, Guangzhou, (N 23°8′, E 113°17′), soil, dried holotype HMAS 352277, ex-type culture CGMCC 3.24163 = GZUIFR 22.394. GenBank accession numbers: OP454339 (ITS), OP547315 (*tub2*), OP454369 (LSU), and OP491393 (*RPB2*).

Micromorphology: Somatic hyphae hyaline, smooth-walled, short septate, 1.5–4 μm wide. Conidiophores reduced to conidiogenous cells. Conidiogenous cells arising laterally from the hyphae, phialidic, solitary, smooth-walled, swollen near the base, cylindrical or flask-shaped, tapering to a narrow neck, 6–21 × 2–3.5 μm. Conidia one-celled, formed in basipetal chains, sometimes apical capitate, hyaline, smooth, fusiform, ellipsoidal or ovoid, 4.5–7 × 2.5–3 μm. Sexual morph not observed.

Culture characteristics: Colonies on PDA reaching 35–40 mm diam after 7 d at 35 °C, irregular in the margin, flat, dense, fluffy, pale wheat, reverse dark, edge pale yellow. Colonies on OA reaching 60–62 mm diam after 7 d at 35 °C, irregular in the margin, flat, dense, fluffy, white, reverse pale yellow.

Geographical distribution: China.

Additional materials examined: China, Guangdong, Guangzhou, (N 23°8′, E 113°17′), soil, and our other living strains: GZUIFR 22.395, GZUIFR 22.396. GenBank accession numbers: OP454340 (ITS), OP547315 (*tub2*), OP454369 (LSU), OP491393 (*RPB2*) and OP454340 (ITS), OP547316 (*tub2*), OP454370 (LSU), and OP491394 (*RPB2*).

Notes: Phylogenetically, this species is closely related to *A. ellpsoidea* and *A. levis* (Figure 1); however, the sequence similarities of the ITS between these species were 98.6% and 97.7%, respectively. Morphologically, the conidia of *A. guangdongensis* are sometimes apical capitate. This feature is similar to *A. multiforma*, but *A. guangdongensis* can be distinguished by its shorter conidial chains.

**Figure 5:**
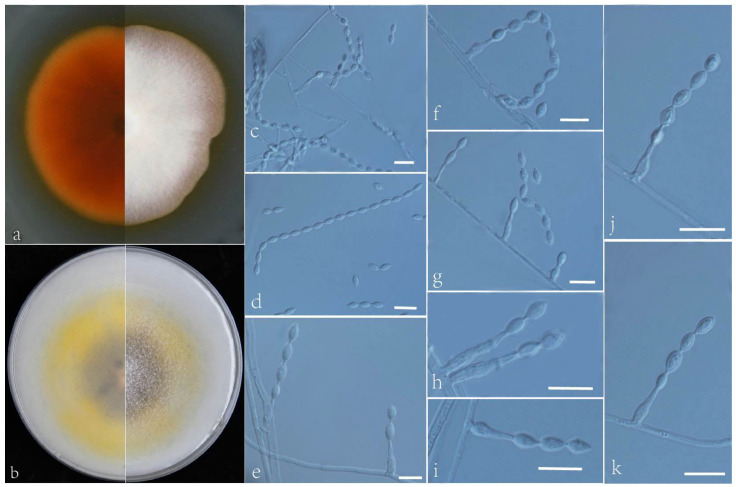
*Acrophialophora longicatenata* (ex-type CGMCC 3.24169). (**a**,**b**) The reverse and obverse colonies on PDA and OA after 7 d at 35 °C. (**c**–**k**) Hyphae, phialidic conidiogenous cells, and conidia. Scale bars: (**c**–**k**) = 10 μm.

***Acrophialophora longicatenata*** L. Peng & Y.F. Han sp. nov. Figure 5

MycoBank: 845783

Etymology: Refers to longer conidial chain.

Holotype: China, Yunnan, Dai Autonomous Prefecture of Xishuangbanna (N 21°40′, E 101°50′), soil, dried holotype HMAS 255325, ex-type culture CGMCC 3.24169 = GZUIFR 22.412. GenBank accession numbers: OP454357 (ITS), OP547309 (*tub2*), OP454378 (LSU), and OP834081 (*RPB2*).

Micromorphology: Somatic hyphae hyaline, smooth-walled, septate, 1–4 μm wide. Conidiophores simple, phialidic, single, arising laterally from the hyphae, swollen basal portion, obclavate or flask-shaped, tapering into a distinct neck, 5–20 × 2–3.5 μm. Conidia one-celled, formed in basipetal chains, often more than 10 conidia, smooth, fusiform or ellipsoidal, 3–6 × 2–4 μm. Sexual morph not observed.

Culture characteristics: Colonies on PDA reaching 55–56 mm diam after 7 d at 35 °C, mostly irregular in the margin, flat, center dense and fluffy and edge sparse, white, reverse sandy brown. Colonies on OA reaching 60–62 mm diam after 7 d at 35 °C, center brown, edge bright yellow, white floccose due to aerial mycelium, reverse center brown, edge bright yellow.

Geographical distribution: China.

Additional material examined: China, Yunnan, Dai Autonomous Prefecture of Xishuangbanna (N 21°40′, E 101°50′), soil, and our other living strains: GZUIFR 22.413, GZUIFR 22.414. GenBank accession numbers: OP454358 (ITS), OP547310 (*tub2*), OP454379 (LSU), OP834082 (*RPB2*) and OP454359 (ITS), OP547311 (*tub2*), OP454380 (LSU), and OP834083 (*RPB2*).

Notes: Phylogenetically, *Acrophialophora longicatenata* is closely related to *A. angustiphialis* (Figure 1); however, the sequence similarity of the *tub2* between the two species was 97.8%. Morphologically, *A. longicatenata* and *A. angustiphialis* have smooth, ellipsoidal, and fusiform conidia [17]. However, they can be distinguished by their conidial chains; the conidial chain of *A. longicatenata* is usually more than 10 conidia.

**Figure 6:**
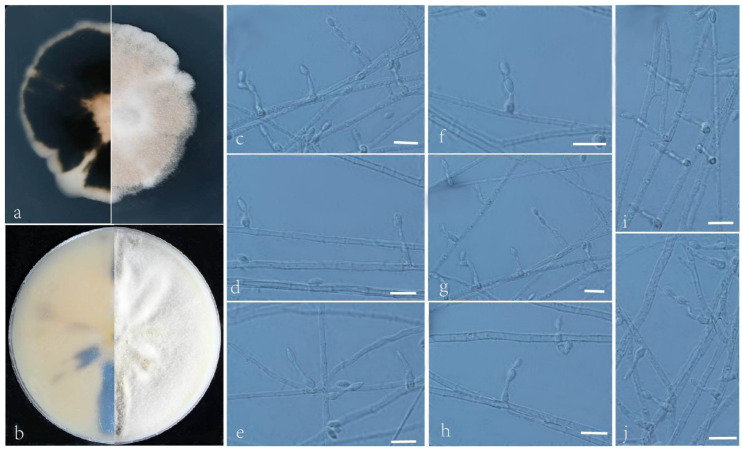
*Acrophialophora minuta* (ex-type CGMCC 3.24165). (**a**,**b**) the reverse and obverse colonies on PDA and OA after 7 d at 35 °C. (**c**–**j**) Hyphae, phialidic conidiogenous cells, and conidia. Scale bars: (**c**–**j**) = 10 μm.

***Acrophialophora minuta*** L. Peng & Y.F. Han sp. nov. Figure 6

MycoBank: 845778

Etymology: Refers to its smaller conidia and phialides.

Holotype: China, Fujian, Fuzhou, Fujian Normal University (N 26°03′, E 119°21′), soil, Y. F. Han, dried holotype HMAS 352278, ex-type culture CGMCC 3.24165 = GZUIFR 22.400. GenBank accession numbers: OP454342 (ITS), OP547318 (*tub2*), OP454366 (LSU), and OP880249 (*RPB2*).

Micromorphology: Somatic hyphae hyaline, smooth-walled, septate, 1–4 μm wide. Conidiophores single, phialidic, arising laterally from the hyphae, swollen basal portion, tapering into a distinct neck, 4–16 × 2–3 μm. Conidia one-celled, formed in basipetal chains, short conidia chain, often composed of one to three conidia, hyaline, smooth, fusiform or ellipsoidal, 4–6 × 2–3 μm. Sexual morph not observed.

Culture characteristics: Colonies on PDA reaching 45–50 mm diam after 7 d at 35 °C, irregular in the margin, flat, dense, fluffy, white to pale brown, reverse center to edge, pale yellow, dark to beige. Colonies on OA reaching 80–82 mm diam after 7 d at 35 °C, raised, dense, fluffy, white, reverse pale yellow and dark.

Geographical distribution: China.

Additional materials examined: China, Fujian Province, Fuzhou City, Fujian Normal University (N 26°03′, E 119°21′), soil, and our other living strains: GZUIFR 22.401, GZUIFR 22.402. GenBank accession numbers: OP454343 (ITS), OP547319 (*tub2*), OP454367 (LSU), OP880250 (*RPB2*) and OP454344 (ITS), OP547320 (*tub2*), OP454368 (LSU), and OP880251 (*RPB2*).

Notes: Phylogenetically, this species is closely related to *A. multiforma* (Figure 1); however, the sequence similarities of the ITS between the two species were 98.5%. Morphologically, *A. minuta* can be distinguished from *A. multiforma* by its smaller phialides (4–15 × 2–3 μm vs. 5–25 × 2–3.5 μm) and conidia (4–5.5 × 2–3 μm vs. 4.5–7 × 2–3.5 μm).

**Figure 7:**
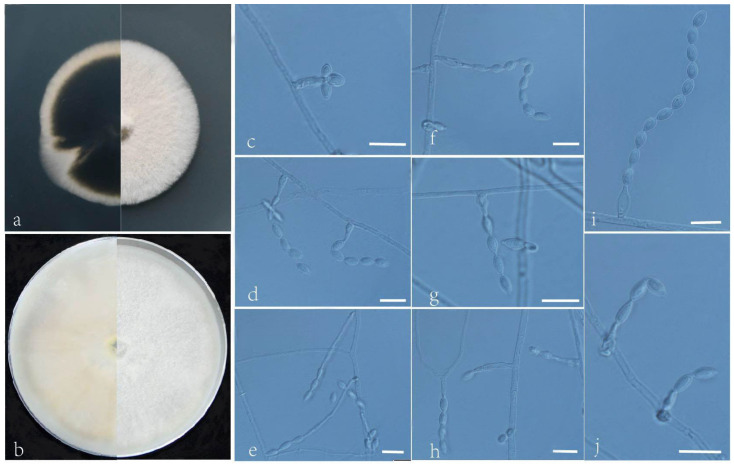
*Acrophialophora multiforma* (ex-type CGMCC 3.24164). (**a**,**b**) The reverse and obverse colonies on PDA and OA after 7 d at 35 °C. (**c**–**j**) Hyphae, phialidic conidiogenous cells, and conidia. Scale bars: (**c**–**j**) = 10 μm.

***Acrophialophora multiforma*** L. Peng & Y.F. Han sp. nov. Figure 7

MycoBank: 845779

Etymology: Refers to the variety of phialides.

Holotype: China, Yunnan, Baoshan, Tengchong County (N 24°50′, E 98°39′), soil, dried holotype HMAS 255321, ex-type culture CGMCC 3.24164 = GZUIFR 22.397. GenBank accession numbers: OP454336 (ITS), OP547321 (*tub2*), OP454360 (LSU), and OP880243 (*RPB2*).

Micromorphology: Somatic hyphae hyaline, smooth-walled, septate, 1–4 μm wide. Conidiophores absent or reduced to conidiogenous cells. Conidiogenous cells arising laterally from the hyphae, phialidic, solitary, smooth-walled, swollen near the basal portion, flask-shaped, obclavate, tapering to a narrow neck, sometimes awl-shaped or linear, 5–25 × 2–3.5 μm. Conidia one-celled, formed in basipetal chains, long chain, sometimes apical capitate, hyaline, smooth, fusiform or ellipsoidal, 4.5–7 × 2–3.5 μm. Sexual morph not observed.

Culture characteristics: Colonies on PDA reaching 44–46 mm diam after 7 d at 35 °C, mostly regular in the margin, flat, dense, fluffy, beige, reverse center dark, edge beige. Colonies on OA reaching 78–80 mm diam after 7 d at 35 °C, irregular in the margin, flat, dense, fluffy, white, reverse pale yellow.

Geographical distribution: China.

Additional materials examined: China, Yunnan, Baoshan, Tengchong County (N 24°50′, E 98°39′), soil, and our other living strains: GZUIFR 22.398, GZUIFR 22.399. GenBank accession numbers: OP454337 (ITS), OP547322 (*tub2*), OP454361(LSU), OP880244 (*RPB2*) and OP454338 (ITS), OP547323 (*tub2*), OP454362 (LSU), and OP880245 (*RPB2*).

Notes: This species is characterized by the variety of phialides and conidia, which are sometimes apical capitate. This feature distinguishes the species from the other species of *Acrophialophora*.

**Figure 8:**
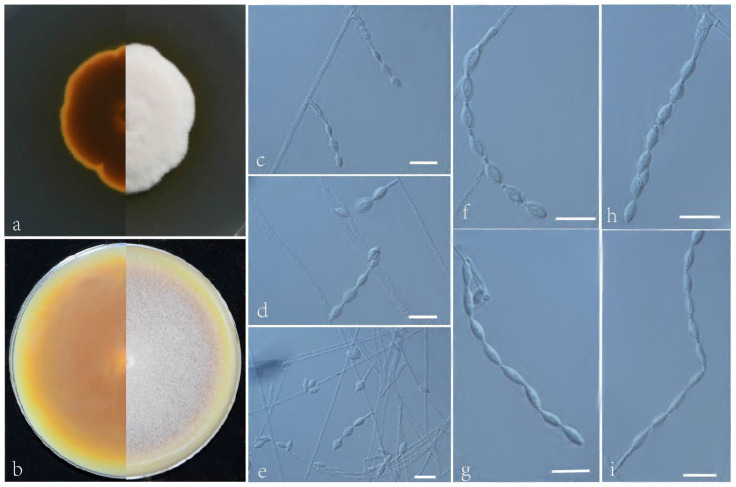
*Acrophialophora rhombica* (ex-type CGMCC 3.24170). (**a**,**b**) I reverse and obverse colonies on PDA and OA after 7 d at 35 °C. (**c**–**i**) Hyphae, phialidic conidiogenous cells, and conidia. Scale bars: (**c**–**i**) = 10 μm.

***Acrophialophora rhombica*** L. Peng & Y.F. Han sp. nov. Figure 8

MycoBank: 845784

Etymology: Refers to nearly rhombic conidia.

Holotype: China, Hainan, Sanya, (N 18°20′, E 109°50′), soil, dried holotype HMAS 352281, ex-type culture CGMCC 3.24170 = GZUIFR 22.415. GenBank accession numbers: OP454354 (ITS), OP731571 (*tub2*), OP454381 (LSU), and OP834084 (*RPB2*).

Micromorphology: Somatic hyphae hyaline, smooth-walled, septate, 1–4.5 μm wide. Conidiophores absent or simple, phialidic, single, arising laterally from the hyphae, with an awl-shaped swollen basal portion, tapering gradually or abruptly to a narrow neck, 5–18 × 1.5–4 μm. Conidia one-celled, formed in basipetal chains, long chain, smooth, fusiform, long ellipsoidal or rhombic, 5–9 × 2–5 μm. Sexual morph not observed.

Culture characteristics: Colonies on PDA reaching 35–40 mm diam after 7 d at 35 °C, mostly irregular in the margin, thick, spongy, cream, reverse brown, edge orange. Colonies on OA reaching 78–80 mm diam after 7 d at 35 °C, brown, white floccose due to aerial mycelium, reverse yellow-brown.

Geographical distribution: China.

Additional material examined: China, Hainan, Sanya, (N 18°20′, E 109°50′), soil, our other living strains: GZUIFR 22.416, GZUIFR 22.417. GenBank accession numbers: OP454355 (ITS), OP731572 (*tub2*), OP454382 (LSU), OP834085 (*RPB2*) and OP454356 (ITS), OP731573 (*tub2*), OP454383 (LSU), and OP834086 (*RPB2*).

Notes: Morphologically, this species is similar to *Acrophialophora acuticonidiata*; however, it can be distinguished by its rhombic and long ellipsoidal conidia. In addition, the conidia of *A. rhombica* are smooth, while those of *A. acuticonidiata* are rough [17].

**Figure 9:**
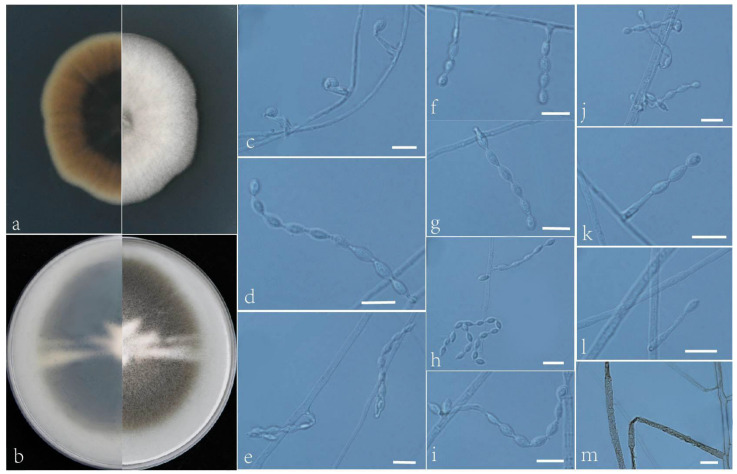
*Acrophialophora yunnanensis* (ex-type CGMCC 3.24168). (**a**,**b**) the reverse and obverse colonies on PDA and OA after 7 d at 35 °C. (**c**–**m**) Hyphae, phialidic conidiogenous cells, and conidia. Scale bars: (**c**–**m**) = 10 μm.

***Acrophialophora yunnanensis*** L. Peng & Y.F. Han sp. nov. Figure 9

MycoBank: 845785

Etymology: Refers to Yunnan Province, where the isolate was collected.

Holotype: China, Yunnan, Dai Autonomous Prefecture of Xishuangbanna (N 21°40′, E 101°50′), soil, dried holotype HMAS 352280, ex-type culture CGMCC 3.24168 = GZUIFR 22.409. GenBank accession numbers: OP454348 (ITS), OP547312 (*tub2*), OP454375 (LSU), and OP880246 (*RPB2*).

Micromorphology: Somatic hyphae hyaline or brown, smooth-walled or rough, septate, 1–4.5 μm wide. Conidiophores absent or usually reduced to conidiogenous cells. Conidiogenous cells, single, phialidic, arising laterally from the hyphae, swollen basal portion, tapering into a distinct neck, awl-shaped, or forming a thin and short neck, 4.5–20 × 2–2.5 μm. Conidia 1-celled, formed in basipetal chains, long chain, smooth, fusiform or ellipsoidal to long ellipsoidal, 3.5–8 × 1.5–4 μm. Sexual morph not observed.

Culture characteristics: Colonies on PDA reaching 35–40 mm diam after 7 d at 35 °C, irregular in the margin, flat, dense, fluffy, light gray, reverse center to edge, dark, golden olive to light yellow. Colonies on OA reaching 65–68 mm diam after 7 d at 35 °C, puce to white, white floccose due to aerial mycelium, reverse dark to white.

Geographical distribution: China.

Additional material examined: China, Yunnan, Dai Autonomous Prefecture of Xishuangbanna (N 21°40′, E 101°50′), soil, and our other living strains: GZUIFR 22.410, GZUIFR 22.411. GenBank accession numbers: OP454349 (ITS), OP547313 (*tub2*), OP454376 (LSU), OP880247 (*RPB2*) and OP454350 (ITS), OP547314 (*tub2*), OP454377 (LSU), and OP880248 (*RPB2*).

Notes: Phylogenetically, this species is closely related to *Acrophialophora rhombica* (Figure 1); however, the sequence similarity of the ITS between the two species was 98.6%, and the sequence similarity of the *RPB2* was 98.3%. Morphologically, it can be distinguished by its conidial shape and somatic hyphae. *A. rhombica* has nearly rhombic conidia as well as hyaline hyphae that are smooth-walled. However, the somatic hyphae of *A. yunnanensis* are sometimes brown and rough-walled.

## 4. Discussion

In this study, the ITS sequences of eight new species were uploaded to preliminary BLAST searches, and the results indicated that they were most closely related to the genus *Acrophialophora*. We then established the phylogenetic tree of *Acrophialophora* and showed that eight new species were indeed located in *Acrophialophora* (Figure 1). The species of the genus *Acrophialophora* were found to form five distinct subclades. Originally, only asexual states were known for the genus *Acrophialophora*, and subsequently, *A. teleoafricana* and *A. jodhpurense*, as one of the subclades with sexual structures, were accommodated in the genus [20]. In addition, there were three *Acrophialophora* species: *A. ampullaris*, *A. ampullaphora*, and *A. berberidis*, which were not discussed in this study due to a lack of sequence support. However, according to the morphological description and illustration of Han et al. and Matushima [14,31,32], *A. ampulliphora* has rough conidia, and *A. ampullaris* has subglobose conidia, which can be distinguished from the eight new species with smooth and fusiform or ellipsoidal conidia, respectively. *Acrophialophora berberidis* is similar to *A. guangdongensis* and *A. multiforma* in having both capitate and catenulate conidia, but the phialide bases of *A. guangdongensis* are cylindrical swollen, of *A. multiforma* are multiform, and of *A. berberidis* are ellipsoidal. Therefore, the three species are different from our eight new species. However, the taxonomic statuses of these three species still need further study.

Some genera of *Chaetomiaceae* have certain morphological similarities to *Acrophialophora*, including *Botryotrichum* and *Humicola*. Some species of *Botryotrichum* and *Humicola* also directly produce phialides and conidial chains on the hyphae. However, these fungi are characterized by simple, short conidiophores and brown subglobose or globose conidia [13]. *Monocillium*, *Acremonium*, and *Phialemonium* are also somewhat similar to these genera in morphology, but they do not belong to *Chaetomiaceae*. *Monocillium* has a similar conidiogenous structure, which is distinguished by the inflated or thickened middle part of the phialide [33]. However, the *Acremonium* species can be distinguished by their typical awl-shaped phialides [34]. *Phialemonium inflatum* is similar in morphology, but it is not thermotolerant [16].

*Chaetomiaceae* includes mostly soilborne cellulose decomposers and thermotolerant opportunistic pathogens [18]. In the same way, almost all species of *Acrophialophora* were proven to be thermotolerant. Studies have shown that some species can produce useful thermostable enzymes. For example, *A. major* and *A. biformis* can produce laccase, and *A. cinerea* and *A. hechuanensis* can produce ligninase and cellulase [10,35,36]. However, Sandoval et al. first studied the clinical significance of *Acrophialophora*, and they considered that the genus is a rare opportunistic human and animal pathogen and includes common species isolated from human clinical samples, such as *Acrophialophora levis* [18]. The study also mentioned that one clinical isolate, FMR 8888, which caused corneal infection, was confirmed as *Acrophialophora fusispora*, while another, FMR 6662, which was isolated from sputum, was reidentified as *Acrophialophora levis*. Therefore, the potential of these fungi in applied research and their pathogenicity to humans need to be further studied.

## Figures and Tables

**Table 1:** Names, strain numbers, and corresponding GenBank accession numbers of the taxa used in the phylogenetic analyses of this study.

Species	Strains	GenBank Accession Numbers			
		ITS	*tub2*	LSU	*RPB2*
*Acrophialophora acuticonidiata*	CGMCC 3.17245	KJ026975	KJ147441	—	—
*Acrophialophora angustiphialis*	CGMCC 3.15258	KJ026972	KJ147438	—	—
*Acrophialophora biformis*	GZDXIFR-H28-1	DQ191963	—	—	—
*Acrophialophora cinerea*	GZDXIFR-H-57-1	DQ243694	KP143110	OP419973	OP886702
*Acrophialophora curticatenata*	GZUIFR-H125-2	EU004811	—	—	—
*Acrophialophora curvata*	GZUIFR 22.406	OP454351	OP547306	OP454363	OP802834
*Acrophialophora curvata*	GZUIFR 22.407	OP454352	OP547307	OP454364	OP802835
*Acrophialophora curvata*	GZUIFR 22.408	OP454353	OP547308	OP454365	OP802836
*Acrophialophora ellipsoidea*	CBS 102.61	MK926786	MK926886	MK926786	MK876748
*Acrophialophora fujianensis*	GZUIFR 22.403	OP454345	OP536984	OP454372	OP820579
*Acrophialophora fujianensis*	GZUIFR 22.404	OP454346	OP536985	OP454373	OP820580
*Acrophialophora fujianensis*	GZUIFR 22.405	OP454347	OP536986	OP454374	OP820581
*Acrophialophora furcata*	GZDXIFR-H-104-1	DQ243695	KP143113	OP456145	OP886703
*Acrophialophora fusispora*	CBS 380.55	MK926788	MK926888	MK926788	MK876750
*Acrophialophora guangdongensis*	GZUIFR 22.394 T	OP454339	OP547315	OP454369	OP491393
*Acrophialophora guangdongensis*	GZUIFR 22.395	OP454340	OP547316	OP454370	OP491394
*Acrophialophora guangdongensis*	GZUIFR 22.396	OP454341	OP547317	OP454371	OP491395
*Acrophialophora hechuanensis*	GZUIFR-H08-1	MK926789	MK926889	MK926789	MK876751
*Acrophialophora jiangsuensis*	GZUIFR HC48.1	KF719171	KP143112	OP456146	OP491392
*Acrophialophora jodhpurensis*	CBS 602.69	MK926790	MK926890	MK926790	MK876752
*Acrophialophora levis*	CBS 484.70	KP233038	KP233044	KM995840	—
*Acrophialophora liboensis*	CGMCC 3.18309	KP192127	KP999978	OP456147	OP886704
*Acrophialophora longicatenata*	GZUIFR 22.412 T	OP454357	OP547309	OP454378	OP834081
*Acrophialophora longicatenata*	GZUIFR 22.413	OP454358	OP547310	OP454379	OP834082
*Acrophialophora longicatenata*	GZUIFR 22.414	OP454359	OP547311	OP454380	OP834083
*Acrophialophora major*	GZUIFR-H57-2	MK926792	MK926892	MK926792	MK876754
*Acrophialophora minuta*	GZUIFR 22.400 T	OP454342	OP547318	OP454366	OP880249
*Acrophialophora minuta*	GZUIFR 22.401	OP454343	OP547319	OP454367	OP880250
*Acrophialophora minuta*	GZUIFR 22.402	OP454344	OP547320	OP454368	OP880251
*Acrophialophora multiforma*	GZUIFR 22.397 T	OP454336	OP547321	OP454360	OP880243
*Acrophialophora multiforma*	GZUIFR 22.398	OP454337	OP547322	OP454361	OP880244
*Acrophialophora multiforma*	GZUIFR 22.399	OP454338	OP547323	OP454362	OP880245
*Acrophialophora nainiana*	CBS 100.60	MK926793	MK926893	MK926793	MK876755
*Acrophialophora rhombica*	GZUIFR 22.415 T	OP454354	OP731571	OP454381	OP834084
*Acrophialophora rhombica*	GZUIFR 22.416	OP454355	OP731572	OP454382	OP834085
*Acrophialophora rhombica*	GZUIFR 22.417	OP454356	OP731573	OP454383	OP834086
*Acrophialophora seudatica*	CBS 916.79	LN736030	LN736032	LN736031	—
*Acrophialophora teleoafricana*	CBS 281.79	MK926795	MK926895	MK926795	MK876757
*Acrophialophora yunnanensis*	GZUIFR 22.409 T	OP454348	OP547312	OP454375	OP880246
*Acrophialophora yunnanensis*	GZUIFR 22.410	OP454349	OP547313	OP454376	OP880247
*Acrophialophora yunnanensis*	GZUIFR 22.411	OP454350	OP547314	OP454377	OP880248
*Chrysocorona lucknowensis*	CBS 727.71 T	MK926813	MK926913	MK926813	MK876773

**Table 2:** The best-fit evolutionary model phylogenetic analyses.

	ITS	LSU	*tub2*	*RPB2*
ML analysis	K3P+G4	F81+F+I	HKY+F+G4	GTR+F+G4
BI analysis	K2P+G4	F81+F+I	HKY+F+G4	GTR+F+G4

## Data Availability

All newly generated sequences were deposited in GenBank (https://www.ncbi.nlm.nih.gov/genbank/, (accessed on 13 September 2022) Table 1). All new taxa were deposited in MycoBank (https://www.mycobank.org/ (accessed on 3 December 2022)).
